# Roles of NRXN1 in neuropsychiatric disorders: from genetic lesion to molecular mechanism

**DOI:** 10.3389/fnins.2026.1808921

**Published:** 2026-05-13

**Authors:** Jiaxiang Liu, Yanxuan Zhang, Ruijia Jin, Yuxin Zhu, Jianrui Chen

**Affiliations:** 1 Queen Mary School, Medical Department, Nanchang University, Nanchang, China; 2 Basic Medical College of Nanchang University, Jiangxi Medical College, Nanchang University, Nanchang, China; 3 Hospital of Stomatology, Jilin University, Changchun, China; 4 Department of Cardiology, Zhongshan Hospital, Fudan University, Shanghai Institute of Cardiovascular Diseases, Shanghai, China

**Keywords:** autism, neurexin, neuropsychiatric disorders, NRXN1, schizophrenia

## Abstract

Numerous neuropsychiatric disorders frequently exhibit overlapping genetic risk factors, implying the molecular basis for their comorbidity. Nevertheless, the pathogenesis of these disorders remains elusive, particularly regarding how genetic variations impair the physiological function of risk genes and contribute to disease phenotypes. Neurexin 1 protein, encoded by *NRXN1* gene, belongs to the neurexin family of presynaptic adhesion molecules. And neurexin 1 is involved in synaptogenesis and the maintenance of synaptic action. Genetic variations of *NRXN1* have been demonstrated to be associated with a spectrum of neuropsychiatric disorders. Herein, this review focuses on the most recent and relevant literature concerning the genetic and molecular mechanisms through which *NRXN1* variants contribute to the pathogenesis of neuropsychiatric disorders, particularly schizophrenia and autism spectrum disorder. Among them, we propose the isoform-dependent excitation-inhibition imbalance hypothesis of *NRXN1* in autism spectrum disorder. And this hypothesis may account for both the elevated and decreased excitation-inhibition ratios observed in diverse individuals with autism spectrum disorder. Moreover, both schizophrenia and autism spectrum disorder involve deletions and alternative splicing of *NRXN1*, offering molecular evidence for their comorbidity. Then, we analyzed and summarized the current research status of *NRXN1* in other neuropsychiatric disorders, including attention-deficit hyperactivity disorder, insomnia, epilepsy, suicide, and depression. Additionally, available limited researches on *NRXN1*-targeted therapeutic strategies and associated pharmacological studies are also incorporated. Finally, we discussed existing challenges in *NRXN1* research within the context of neuropsychiatric disorders and proposed potential avenues to overcome these obstacles.

## 1 Introduction

Neuropsychiatric disorders, characterized by concurrent neurological and psychiatric manifestations, represent the primary global cause of disability throughout all life stages (Falk et al., 2016; Kasem et al., 2018; Feigin et al., 2020). For example, around 2.8% of children are diagnosed with autism spectrum disorder (ASD) (Maenner et al., 2023). In addition to their high prevalence, these disorders arise from complex interactions among genetic, environmental, and lifestyle factors and exhibit marked heterogeneity in cognitive profiles, clinical advancement, and neuropathological features, which present substantial challenges for both prevention and therapeutic intervention.

Synaptic adhesion molecules (SAMs) determine the synaptic architecture and functional characteristics, thereby modulating the computational capacity of neural networks (Hu et al., 2019). Neurexins are type I transmembrane proteins that share homology with other cell-surface molecules harboring Laminin-Neurexin-Sex hormone-binding globulin (LNS) domains (Ushkaryov et al., 1992; Ushkaryov et al., 1994). In neurons, the selective localization of neurexins at synapses defines them as SAMs (Ushkaryov et al., 1992). In addition to neurons, neurexin is broadly expressed in non-neuronal cells, such as glial cells (Uchigashima et al., 2019), pancreatic β-cells (Mosedale et al., 2012), melanotrophs of the pituitary gland (Dudanova et al., 2006) and vascular endothelial cells (Bottos et al., 2009). Neurexins are classified into three genes: *NRXN1–3* in humans and *Nrxn1–3* in mice (Ichtchenko et al., 1996). Mutations in neurexins, especially *NRXN1*, have been demonstrated to be highly correlated with multiple neuropsychiatric disorders, particularly schizophrenia (Marshall et al., 2017; Castronovo et al., 2020) and ASD (Marshall et al., 2008; Montalbano et al., 2024).

*NRXN1*, the sole gene within the 2p16.3 locus, encodes the protein neurexin 1 (NRXN1) (Südhof, 2017). The *NRXN1* gene undergoes extensive alternative splicing at sites SS1–SS6, producing functionally distinct *α* and *β* isoforms that differentially regulate ligand-specific interactions with ligands, including neuroligins (NLGNs) and leucine-rich repeat transmembrane neuronal proteins (LRRTMs) (Südhof, 2017). These trans-synaptic connections critically regulate multiple fundamental synaptic processes, including synaptogenesis, neurotransmission efficiency, and activity-dependent synaptic plasticity (Dean et al., 2003). Furthermore, various alternative splice variants and isoforms of neurexin 1 contribute to multiple physiological and pathological mechanisms, including the synaptic maturation (Nozawa et al., 2022), the imbalance between excitatory and inhibitory signaling (E-I imbalance) (Uzunova et al., 2016), and the frequency of miniature excitatory postsynaptic currents (mEPSC) (Chen et al., 2010).

Given the rapidly accumulating evidence for the roles of *NRXN1* in psychiatric pathogenesis, a review of the current evidence is essential to consolidate existing findings and elucidate its potential utility in future preventive and therapeutic interventions. Accordingly, this review covers recent research on the association between *NRXN1* and neuropsychiatric disorders, with a focus on elucidating its pathophysiological mechanisms in disease pathogenesis.

## 2 Gene organization of *NRXN1-3* and location of NRXN1 protein

**Figure 1:**
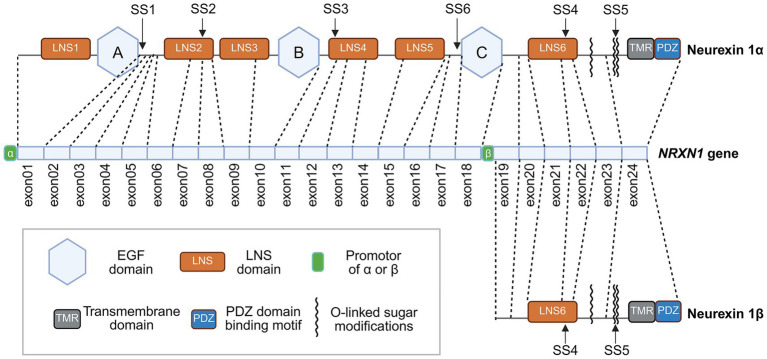
Genomic organization of *NRXN1* gene and structures of its protein isoforms neurexin 1α and neurexin 1β. *NRXN1* gene consists of 24 exons. Dotted lines indicate the correspondence between exons and protein domains. Neurexin 1α is transcribed from the promoter of α located upstream of exon 1, whereas neurexin 1β is transcribed from the promoter of β located downstream of exon 18. The neurexin 1α isoform contains three EGF domains, six LNS domains, a carboxy-terminal glycosylated region, a transmembrane domain, and a PDZ domain-binding motif. Neurexin 1β isoform contains an LNS domain, a carboxy-terminal glycosylated region, a transmembrane domain, and a PDZ domain-binding motif. The positions of SS1-6 are marked in the figure.

Each *NRXN* gene (*NRXN1-3*) encodes two major protein isoforms: the longer NRXNα and the shorter NRXNβ (Ullrich et al., 1995). These two isoforms are transcribed from independent promoters yet share most of their coding sequences (Ullrich et al., 1995). Compared to the NRXN1α isoform, NRXN1β constitutes a truncated isoform generated through transcriptional initiation at a downstream promoter following exon 18 in *NRXN1* or exon 17 in *NRXN*2/3, thereby omitting the initial 1–17 or 18 exon segment characteristics of NRXN1α (Ushkaryov et al., 1994; Treutlein et al., 2014) (Figure 1). Uniquely, the *NRXN1* gene expresses a third isoform, NRXN1γ, from an internal 3′ promoter; this isoform lacks all extracellular domains except for the membrane-proximal sequences (Sterky et al., 2017). The *NRXN1* and *NRXN3* genes rank among the largest in the mammalian genome, spanning over 1 megabase (Mb) in both mice and humans (Rowen et al., 2002; Tabuchi and Südhof, 2002). And *NRXN1* expression increased progressively with age, while *NRXN2* levels remained consistently higher than *NRXN3* across all age groups examined (Harkin et al., 2017).

The *NRXN1* gene, located at the p16.3 region of human chromosome 2, spans 1.1 Mb and encodes the synaptic adhesion protein NRXN1 with 24 exons (Lukacsovich et al., 2019) (Figure 1). NRXN1 protein, encoded by *NRXN1* gene, is the critical synaptic cell adhesion molecule responsible for both synaptic organization and transmission (Reissner et al., 2013). Specifically, NRXN1 promotes the exocytosis of neurotransmitters and serves as a core component of the trans-synaptic assembly that directs the specification, establishment, and maturation of synaptic connections (Missler et al., 2003).

*Nrxn1* is widely expressed in the cerebral cortex and CA1 and CA3 subregions of the hippocampus (Nguyen et al., 2016). The immunoreactivity of *NRXN1* was generally the strongest in the cortical plate, elevated in the ventricular zone with increasing age, but weak in the synaptogenic presubplate and marginal zone (Harkin et al., 2017). Regarding neuron subtypes, *Nrxn1* is consistently and stably expressed in glutamatergic pyramidal principal neurons, GABAergic Lhx6 + interneurons, and GABAergic Prox1 + interneurons, and owns higher level in glutamatergic neurons (Nguyen et al., 2016; Lukacsovich et al., 2019).

*Nrxn1* mRNAs are also expressed in non-neuronal cells, including astrocytes (Zhang et al., 2014; Zhang et al., 2016) and oligodendrocytes (Uchigashima et al., 2019). Moreover, NRXN1 is also present in microglia, the innate immune cells of the central nervous system, which actively monitor neuronal microenvironmental alterations. In the context of ASD, experimental deletion of NRXN1 in microglia has been found to trigger morphological remodeling and elevate pro-inflammatory cytokine release (e.g., interleukin-6), subsequently disrupting neurodevelopmental processes (Bose et al., 2025).

In addition to nervous system, NRXN1*α* is also expressed in pancreatic β-cells and contributes to the docking of insulin-containing secretory granules (Mosedale et al., 2012). Moreover, *Caenorhabditis elegans* neurexin (NRX1) is also expressed in muscle tissue and mediates a retrograde synaptic signal that inhibits neurotransmitter release at neuromuscular junctions (Hu et al., 2012). Furthermore, NRXNα is also expressed in melanotrophs of the pituitary gland, and it contributes to Ca^2+^-triggered exocytosis of secretory granules (Dudanova et al., 2006). Additionally, NRXNβ is also expressed in vascular endothelial cells and is involved in angiogenesis (Bottos et al., 2009).

## 3 Transcriptional regulation and post-transcriptional modifications of *NRXN1*

### 3.1 Isoforms of NRXN1

*NRXN1* gene produces three protein isoforms through diverse promoter-mediated transcription, with NRXN1α and NRXN1β representing the two major isoforms (Reissner et al., 2013). NRXN1α is expressed through a promoter α located upstream of the exon 1 (Treutlein et al., 2014) (Figure 1). Structurally, this isoform features an extracellular region composed of six tandem LNS domains, interspersed with three epidermal growth factor (EGF)-like motifs and a glycosylation site (Missler and Südhof, 1998). Exon 1 encodes the first LNS domain and an EGF-like domain, while exon 18 encodes the third EGF-like domain (Treutlein et al., 2014). Südhof (2017) have previously reviewed that the LNS6 domain interacts with NLGNs, a series of postsynaptic cell adhesion molecules, to form synapses. Additionally, NRXN1 also possesses multiple other domains: an N-terminal signal peptide that directs its secretion and membrane integration; multiple extracellular modules that mediate specific protein–protein interactions; a single transmembrane domain; and a cytoplasmic C-terminal tail that binds intracellular synaptic scaffolds (Reissner et al., 2013). NRXN1α is essential for Ca^2+^-triggered neurotransmitter release (Missler et al., 2003). They organize synaptic vesicle exocytosis by functionally coupling Ca^2+^ channels to the presynaptic machinery (Missler et al., 2003). Moreover, NRXN1α participates in trans-synaptic complex formation through interactions with diverse postsynaptic partners, such as NLGNs (Ichtchenko et al., 1995), LRRTMs (Ko et al., 2009), calsyntenin 3 (Lu et al., 2014), latrophilin (Boucard et al., 2012), and GABAA receptor (Zhang et al., 2010) on the postsynaptic surface, regulating synaptic development, maintenance, and functional plasticity. Several recent breakthroughs regarding the function of NRXN1α are summarized below. From the perspective of neural circuits, Davatolhagh and Fuccillo (2021) observed both *Nrxn1α^+/−^* and *Nrxn1α^−/−^* mice exhibited reduced resultant neuronal firing rate at indirect pathway spiny projection neurons in the dorsal prefrontal cortical-dorsomedial striatal circuit, due to diminished neurotransmitter release. Though exhibiting relatively normal excitatory resultant neuronal firing rate from thalamic inputs to the dorsal medial striatum, *Nrxn1α^+/−^* and *Nrxn1α^+/−^* mice show alterations in the synaptic content of N-methyl-D-aspartate receptors (NMDARs) (Davatolhagh and Fuccillo, 2021). *Nrxn1α* predominantly sustains baseline release probability and synaptic efficacy in the dorsolateral prefrontal cortex and para-fascicular thalamic connections onto dorsomedial striatal spiny projection neurons (Davatolhagh and Fuccillo, 2021). Moreover, *Nrxn1α* depletion in prefrontal-thalamic networks precipitates cognitive dysfunction and impaired decision-making processes, two common symptoms of neuropsychiatric disorders (Hughes et al., 2022).

NRXN1β isoform exhibits predominant expression in mature brain tissue and features a simplified domain architecture, retaining only the sixth LNS domain in NRXN1α, while lacking the multiple LNS domains, EGF-like motifs in NRXN1α (Araç et al., 2007) (Figure 1). Structural studies reveal that the extracellular domain of NRXN1β mediates interactions with NLGN1 (Hamid et al., 2023), GABAA receptor (Zhang et al., 2010), latrophilin (Boucard et al., 2012) and cerebellins (CBLNs)-GluDs (Uemura et al., 2010). NRXN1β-induced postsynaptic development exhibits PI3K/AKT pathway dependency (Jiang et al., 2021). Disruption of PI3K/AKT cascade markedly reduces excitatory mEPSC frequency, underscoring its essential role in synaptic maturation (Jiang et al., 2021).

NRXN1γ, encoded by an internal 3′ promoter, represents a truncated transcript that lacks key extracellular domains (Yan et al., 2015). Several studies in *Caenorhabditis elegans* have demonstrated the critical role of the γ transcript in promoting and stabilizing presynaptic assemblies (Kurshan et al., 2018; Frankel et al., 2025). NRX1γ is essential for the assembly of active zone components, recruitment of synaptic vesicles, and clustering of calcium channels at release sites, thereby facilitating synaptic transmission (Kurshan et al., 2018). Moreover, NRX1γ localizes to presynapses independently of transsynaptic interactions and stabilizes presynapses against elimination (Frankel et al., 2025).

### 3.2 Splice variants of NRXN1

*NRXN1* harbors six alternative splicing sites (SS1 to SS6) that generate distinct splice variants (Lukacsovich et al., 2019) (Figure 1). Among these, studies on the SS1 variant remain limited, whereas most published research focused on the SS4 and SS5 variants. SS2 is critically involved in mediating neurexophilin (NXPH)-NRXN interactions. The binding interface spans the jelly-roll β-sandwich structure of the NRXN1 LNS2 domain into NXPH1 (Wilson et al., 2019). The SS2A insert of LNS2 reinforces this molecular interface, thereby increasing the binding affinity between LNS2 and NXPH1 (Wilson et al., 2019). Moreover, dystroglycan (DAG)-α engages with both the LNS2 and LNS6 domains of NRXNα in the absence of inserts at splice sites SS2 or SS4, predominantly via glycans attached to its mucin region (Reissner et al., 2014). Surprisingly, DAGα binding to LNS2 inhibits NLGNs interaction with LNS6, regardless of the presence or absence of a splice insert at SS4 (Reissner et al., 2014).

Regarding SS3 splicing, activation of cultured cortical neurons elicits a marked, Ca^2+^-dependent exclusion of the *Nrxn2* SS3 insert (Rozic-Kotliroff and Zisapel, 2007; Rozic et al., 2013). Activity-dependent regulation of neurexin splicing at SS3 contributes to fear learning (Rozic et al., 2011), memory formation (Rozic et al., 2011), and the control of diurnal rhythms (Shapiro-Reznik et al., 2012).

Under normal physiological conditions, the alternative splicing of *NRXN1* gene produces two canonical splice variants: SS4 and SS5. The SS4 + variant selectively interacts with CBLN1/2/4. This interaction facilitates the assembly of the NRXN1qSS4 + -CBLN-GluD1/2 tripartite complex, which potentiates NMDAR-dependent postsynaptic currents by 60% (Dai et al., 2021). Moreover, the SS4- variant interacts with dystroglycans, CIRL/latrophilins and LRRTMs (Siddiqui et al., 2010; Tromp et al., 2021). Studies of dopamine neurons reveal that their characteristic axonal arborization, predominantly featuring non-synaptic terminals, depends critically on the synaptic organizing proteins NRXN1α^SS4-^ and NLGN1^A + B^ for proper synapse formation (Ducrot et al., 2021).

The SS5 splice variant is critically involved in modulating protein stability. The exclusion of SS5 markedly elevates protein abundance and enhances synaptic transmission efficacy through augmented heparan sulfate modification, resulting in a 2.3-fold increase in protein expression (Lu et al., 2023).

SS4 and SS5 cooperatively modulate the synaptic activity of glutamatergic and GABAergic neurons. The *NRXN1+/−* with a 3′-deletion (eliminating SS4 and SS5 sites) resulted in a concurrent decrease in the frequency of spontaneous excitatory postsynaptic currents (sEPSCs) in glutamatergic neurons and an increase in the frequency of spontaneous inhibitory postsynaptic currents (sIPSCs) in GABAergic neurons (Fernando et al., 2025). This indicates a reduction in excitatory synaptic activity and an enhancement of inhibitory synaptic activity (Fernando et al., 2025).

SS6 acts as a molecular hinge in the NRXN1α (Liu et al., 2018). Small-angle X-ray scattering coupled with electron tomography demonstrated that incorporation of the SS6 relieves the restricted conformational flexibility between the L5 and L6 domains (Liu et al., 2018).

## 4 Physiological functions of NRXN1 protein in the nervous system

**Figure 2:**
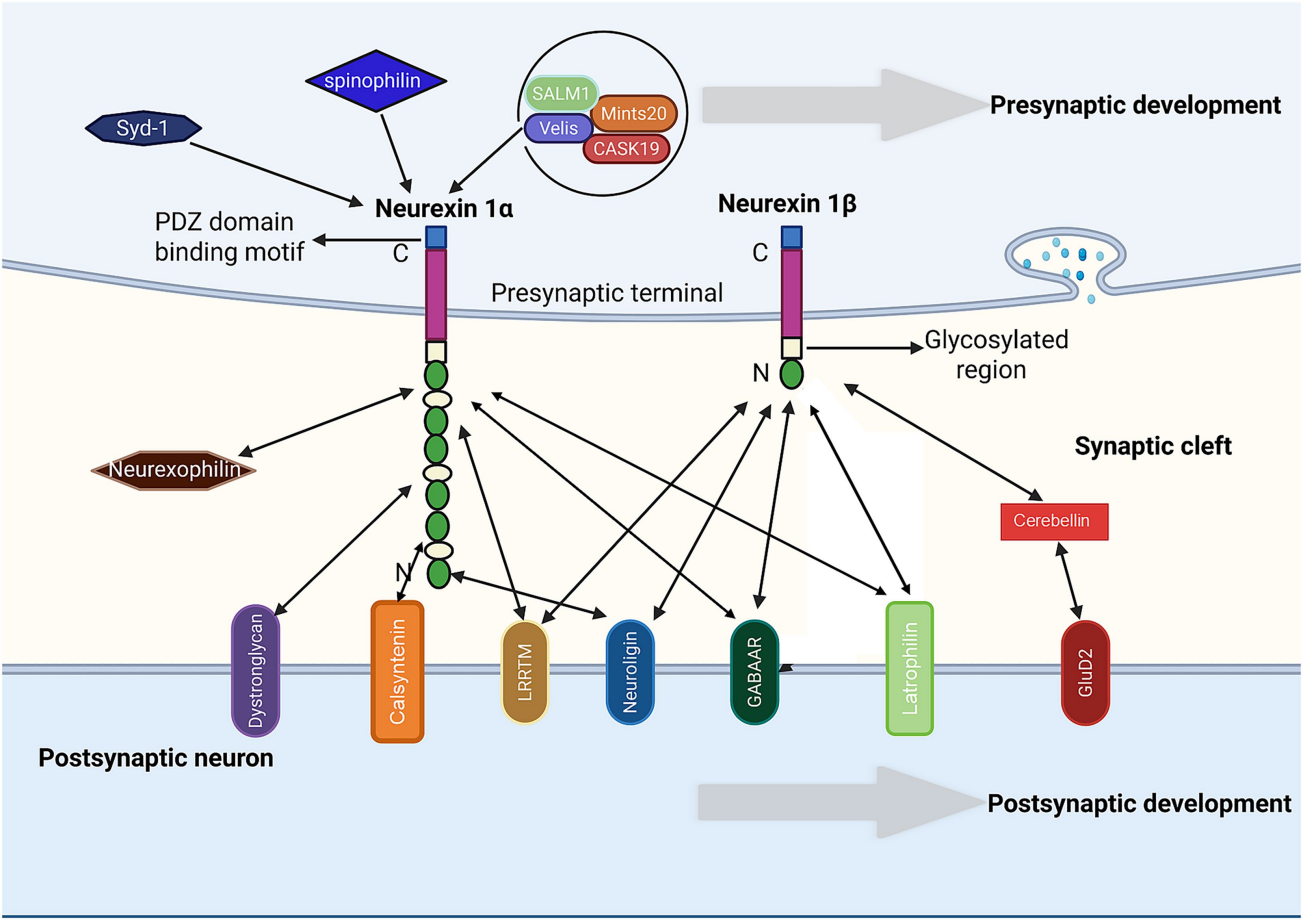
Molecular interactions between main neurexin isoforms and corresponding ligands in terms of synapse development/function. The neurexin 1α and neurexin 1β interact with a series of synaptic ligands to propel the assembly of functional presynaptic and postsynaptic structures.

As an example of SAM, NRXN1 modulates presynaptic neurotransmitter release, and propels the assembly of functional presynaptic structures via trans-synaptic binding to postsynaptic ligands, such as *δ*-type ionotropic glutamate receptors (GluD) (Cortés et al., 2023). In turn, the functional deficiency of NRXN1 results in significant synaptic deficits and disrupted neuronal signaling pathways (Jeon et al., 2021). Molecular interactions between main NRXN1 isoforms (NRXN1α and NRXN1β) and corresponding synaptic ligands are outlined in Figure 2. Intracellularly, the C-terminal region of NRXN1 harbors a presynaptic PDZ-binding motif that mediates specific interactions with the cytoplasmic scaffolding protein CASK (calcium/calmodulin-dependent serine protein kinase) (Pan et al., 2021). This molecular interaction drives the formation of dynamically organized nanoclusters within the presynaptic active zone, the structural arrangement critical for preserving synaptic spatial organization and functional stability (Luo et al., 2021). The PDZ-binding motif of NRXN1 mediates interactions with multiple presynaptic proteins, including Spinophilin, Syd-1, Cbln1, and SALM1, which collectively orchestrate synaptic architecture and function. Spinophilin governs active zone formation and presynaptic release site organization (Muhammad et al., 2015), while Syd-1 drives terminal differentiation and maturation (Owald et al., 2010). Structural studies reveal that Mint20 and CASK19 similarly engage this motif to modulate synaptic vesicle positioning and exocytosis (Hu et al., 2019). Notably, deletion of SALM1 in hippocampal neurons disrupts NRXN1β/NLGN1-dependent excitatory synapse formation, concomitantly impairing vesicle clustering, synaptic transmission efficiency, and release properties (Brouwer et al., 2019). These molecular interactions facilitate synaptic vesicle stabilization and exocytosis, collectively maintaining presynaptic membrane functionality. Beyond direct protein–protein interactions, heparan sulfate modification is also critical for the synaptogenesis function of NRXN1. Mice with heparan sulfate deficiency on NRXN1 demonstrate decreased survival, as well as structural and functional deficits at central synapses (Zhang et al., 2018). Notably, heparan sulfate directly interacts with the postsynaptic partners NLGNs and LRRTMs, revealing a dual binding mechanism that involves both intrinsic glycan and protein domains (Zhang et al., 2018). Given that heparan sulfate chains can also bind numerous ligands, heparan sulfate modification contributes to the functional diversity of NRXN1 (Zhang et al., 2018).

The precise mechanism by which NRXN1 contributes to synaptogenesis has not yet been fully determined. NLGN acts as the canonical ligand for NRXNβ-NRXN in mediating synaptogenesis. Further research demonstrated that NLGN can also interact with NRXNα, an interaction governed by alternative splicing at SSB in NLGN1 and SS4 in NRXNs (Scheiffele et al., 2000). Isoforms of NLGN1 that bind only NRXNβ potently drive synapse assembly, while those capable of associating with both NRXNα and NRXNβ facilitate synapse expansion with greater efficacy (Boucard et al., 2005).

In addition to NLGN, LRRTMs also play a role in synaptogenesis. Uniquely, LRRTM2 induces excitatory synapses by binding to neurexin variants lacking SS4 (Ko et al., 2009). During early synapse development, depletion of either NLGNs or LRRTMs results in significant decreases in AMPA- and NMDA receptor-mediated synaptic currents, indicating functional redundancy between the two families (Soler-Llavina et al., 2011). After synapse maturation, LRRTMs do not alter excitatory transmission, whereas NLGN1 maintains NMDA receptor-mediated signaling (Soler-Llavina et al., 2011).

Recently, cerebellin-1 has been found to be involved in the synaptogenesis of NRXN1. In the initial phase of synaptogenesis, energetically favorable yet functionally inactive cis-interactions between neurexins and their cognate ligands would preferentially form within the same neuronal membrane (Wang et al., 2021). Employing olfactory bulb mitral-granule cell dendro-dendritic synapses as a model system, Wang et al. demonstrate that NRXN1-NLGN1 binding inhibits such cis interactions (Wang et al., 2021). During this process, cerebellin-1, an extracellular scaffolding protein, competitively interferes with cis-oriented NRXN1-NLGN1 complexes, consequently maintaining the synaptogenesis (Wang et al., 2021). Notably, cerebellin-1 and NRXN1β establish trans-synaptic connections in delta glutamate receptors (Carrillo et al., 2021). These binding partners stabilize the extracellular architecture of GluD2 subtype of delta receptor, thereby facilitating them function as synaptic ionotropic neurotransmitter-gated channels (Carrillo et al., 2021). A recent investigation using *Drosophila* mushroom body Kenyon cells found that Nrx1 regulates sequential memory through specialized plasticity modules, which coordinates the structural reorganization of *de novo* synaptogenesis (Turrel et al., 2024). Additionally, NRXN1 is involved in modulating the clustering process in synaptic vesicle cycling (John et al., 2021). The disfunction of synaptic vesicle cycling is the pathogenesis of synaptic vesicle cycling disorders, a neurodevelopmental disorder (John et al., 2021).

NRXN1 modulates the function of multiple brain regions, including medial and dorsal prefrontal cortical, para-fascicular thalamic nucleus and dorsal medial striatum. NRXN1α sustains baseline release probability and synaptic efficacy in the dorsolateral prefrontal cortex and para-fascicular thalamic connections onto dorsomedial striatal spiny projection neurons (Davatolhagh and Fuccillo, 2021). Moreover, the medial prefrontal cortex, a critical brain region mediating higher-order cognitive functions, has been implicated in neuropsychiatric disorders through its regulation of executive processes (Han et al., 2022). *Nrxn1* knockdown in the medial prefrontal cortex of rats results in concurrent neurodevelopmental abnormalities, manifesting as impaired dendritic arborization and distinct behavioral phenotypes characterized by social dysfunction and heightened anxiety-like responses (Wu et al., 2023).

## 5 *NRXN1* mutations in neuropsychiatric disorders

### 5.1 Psychiatric disorders

#### 5.1.1 Schizophrenia

Schizophrenia represents a chronic and severe psychiatric disorder characterized by substantial heritability, affecting approximately 1% of the global population (Faden and Citrome, 2023). This complex psychiatric condition manifests through a series of symptoms: positive symptoms manifesting as delusions and hallucinations, negative symptoms including avolition and anhedonia, as well as cognitive dysfunction (Faden and Citrome, 2023).

A genome-wide association studies (GWAS) involving 41,321 participants has identified copy number variations (CNVs) at eight genomic loci as significant risk factors for schizophrenia, among which the 2p16.3 locus containing *NRXN1* is particularly prominent (Marshall et al., 2017). Another GWAS revealed six susceptibility loci associated with ultra-high risk for schizophrenia in Han Chinese populations, including *NRXN1* (Wang et al., 2024). These loci exhibited significantly elevated expression levels in ultra-high risk individuals and higher conversion rates to schizophrenia within a two-year follow-up period compared to high-risk controls (Wang et al., 2024). A new two-stage analytical strategy integrating real-time quantitative PCR (RT-qPCR) screening with chromosomal microarray analysis demonstrates the functional impact of *NRXN1* CNVs in schizophrenia pathogenesis and supports their potential utility and clinical application as diagnostic biomarkers for this psychiatric disorder (Cheng et al., 2021). The studies investigating the impact of *NRXN1* on schizophrenia are indicated in Table 1. A recent large-scale genomic survey of somatic CNVs identified recurrent somatic deletions encompassing exons 1–5 of the *NRXN1* gene in five out of 12,834 schizophrenia cases (Maury et al., 2023). Subsequent Hi-C chromatin interaction analysis demonstrated that 5′ deletion in *NRXN1* may lead to the formation of aberrant, allele-specific chromatin loops between a putative cryptic promoter and distal non-coding regulatory elements (Maury et al., 2023).

**Table 1:** Summary of studies investigating the impact of NRXN1 mutations in schizophrenia.

Target mutation	Case sample	Control sample	Model/method	Effects/alterations	References
Deletion	12,834		Hi-C chromatin interaction analysis	Gene expression: 5′ deletions in *NRXN1* forms aberrant, allele-specific chromatin loops	Maury et al. (2023)
Deletion	4	4	Brain organoids from iPSCs of schizophrenia patients carrying *NRXN1* deletions	Synaptic activity:↓glutamatergic and GABAergic neuronal maturation;↓synaptic transmission, spontaneous and synchronous synaptic activity	Sebastian et al. (2023)
Deletion	3	3	Neurons model from iPSCs of schizophrenia patients carrying heterogenous *NRXN1* deletions	Synaptic activity:↓spontaneous synaptic events, synaptic responses and synaptic paired-pulse depression; ↑ expression of *CASK*	Pak et al. (2021)
Methylation	50		Sanger sequencing for blood samples of patients with treatment-resistant schizophrenic	Gene expression:↑*NRXN1* methylation after THC consumption, indicating *NRXN1* methylation may contribute to schizophrenia	Jahn et al. (2021)
Methylation	103	105	Genome-wide DNA methylation profiling in 5 EWAS database.	Gene expression: Methylated *NRXN1* are predicted targets of MIR-137, a microRNA in schizophrenia pathogenesis	Forero and González-Giraldo (2020)
Methylation	1,408		Genome-wide DNA methylation profiling of 1,408 human brain samples from three databases.	Gene expression: *NRXN1* contribute to the sexually dimorphic susceptibility of neuropsychiatric disorders by sex-specific methylation modifications	Xia et al. (2021)
Anti-NRXN1α autoantibodies	387	362	Serum and cerebrospinal fluid from schizophrenia patients	Synaptic activity: ↓mEPSC frequency and dendritic spine/synapse density Behavior: Schizophrenia behavior	Shiwaku et al. (2023)

↑: increase; ↓: decrease; Genetic association studies have been marked in blue.

**Figure 3:**
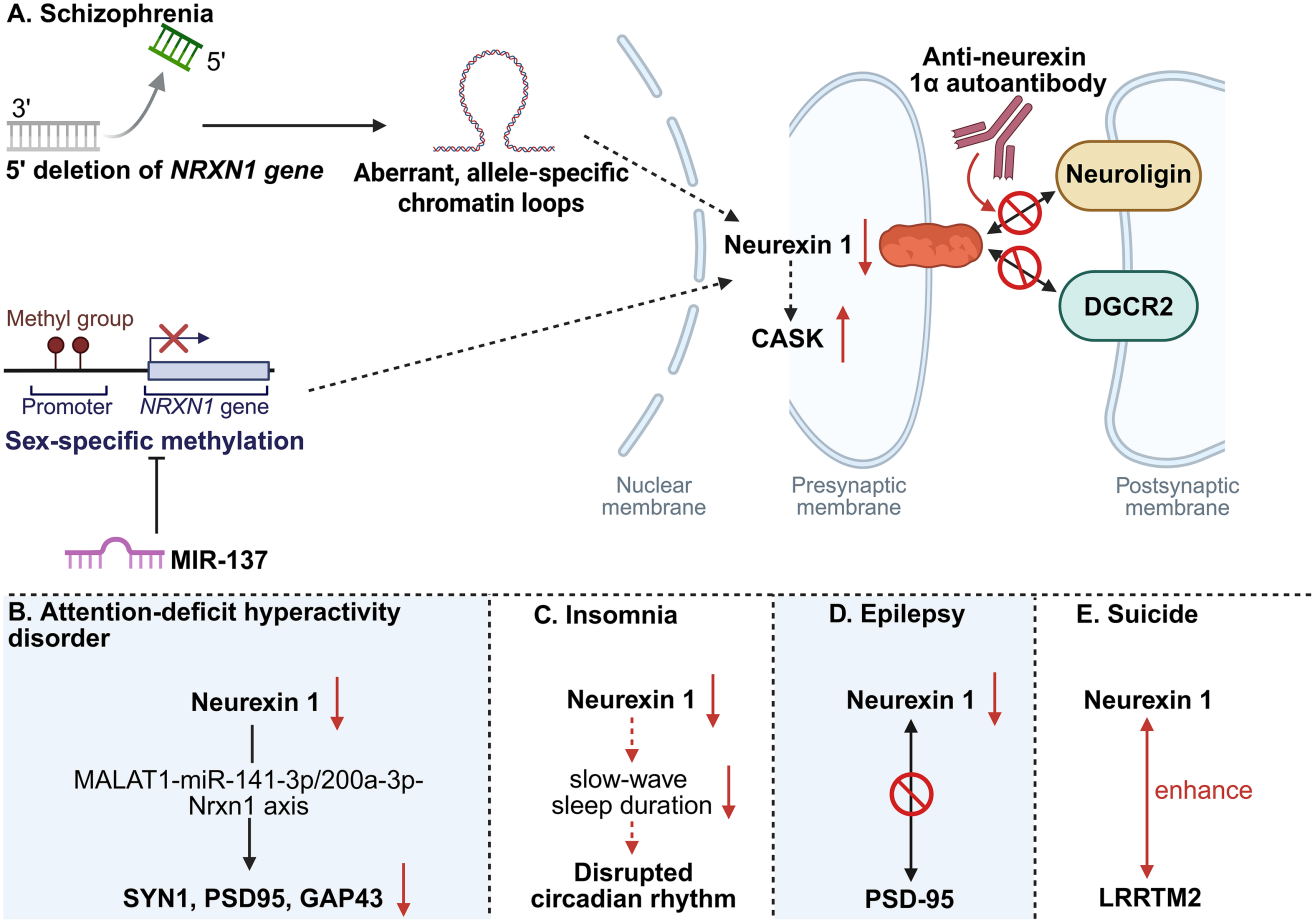
Impacts of mutated *NRXN1* in schizophrenia, attention-deficit hyperactivity disorder, insomnia, epilepsy and suicide. In schizophrenia **(A)**, 5′ deletions in the *NRXN1* gene contribute to the formation of aberrant, allele-specific chromatin loops, leading to the downregulation of neurexin-1 protein. This downregulation can also be caused by sex-specific methylation of the *NRXN1* locus, a process modulated by the miRNA MIR-137. The resulting deficit in neurexin 1 impairs its critical interactions with binding partners, such as neuroligin and/or DGCR2, thereby decreasing mEPSC frequency and dendritic spine/synapse density. Concomitantly, the decrease in neurexin-1 triggers a secondary upregulation of CASK. Moreover, the pathological cascade is potentiated by the presence of anti-neurexin 1α antibodies, which are specifically detected in schizophrenia patients and further disrupt the neurexin 1–neuroligin interaction. In attention-deficit hyperactivity disorder **(B)**, neurexin 1 deficiency mediates the downregulation of postsynaptic molecules (SYN1, PSD95, and GAP43) via the MALAT1-miR-141–3p/200a-3p-Nrxn1 axis. In insomnia **(C)**, reduced neurexin-1 levels are associated with shortened slow-wave sleep duration, ultimately contributing to circadian rhythm disruption. In epilepsy **(D)**, the deficit impairs the specific interaction between neurexin 1 and PSD-95. In suicide **(E)**, the binding affinity between neurexin-1 and LRRTM2 is enhanced.

The impacts of *NRXN1* in schizophrenia are demonstrated in Figure 3A. A systematic review through induced pluripotent stem cells (iPSC) and animal models reveals that among CNVs of neurexin 1, deletions of the neurexin 1 gene represent one of the most prevalent single-gene mutation identified in schizophrenia pathogenesis (Shan et al., 2024). Using cortical brain organoids derived from a heterogeneous cohort of schizophrenia patient-derived iPSC, Sebastian et al. demonstrated that *NRXN1* deletions disrupt neurodevelopmental processes as early as 3 weeks post-differentiation (Sebastian et al., 2023). These deletions were found to impair both glutamatergic and GABAergic neuronal maturation through dysregulation of alternative splicing mechanisms, ubiquitin-proteasome pathways and synaptic communication networks (Sebastian et al., 2023). At the functional level, *NRXN1* deficiencies reduced synaptic transmission, spontaneous and synchronous synaptic activity, indicating synaptic dysfunction (Pak et al., 2021; Sebastian et al., 2023). Mechanistically, *NRXN1* deficiency was associated with elevated expression of CASK, an intracellular binding partner of NRXN1, suggesting a potential compensatory mechanism in response to synaptic impairment (Pak et al., 2021). As an extracellular cell adhesion binding ligand of NRXN1, DGCR2 is critically involved in dendritic spine development and synaptic plasticity (Ren et al., 2023). This molecular interaction between NRXN1 and DGCR2 contributes substantially to the structural and functional integrity of dendritic spines, which represent the principal postsynaptic sites for excitatory transmission in pyramidal neurons (Ren et al., 2023). Notably, the 22q11.2 genomic region containing DGCR2 is frequently deleted in 22q11.2 deletion syndrome, a genetic condition potentially linked to schizophrenia, suggesting that impaired NRXN1-DGCR2 signaling may contribute to the synaptic pathology observed in schizophrenia (Shifman et al., 2006).

Recent studies demonstrated that DNA methylation alterations on the *NRXN1* gene may contribute to the pathogenesis of schizophrenia. Tetrahydrocannabinol (THC), the primary psychoactive component of cannabis, constitutes a significant etiology of schizophrenia (Jahn et al., 2021). THC can reverse the normal hypomethylation state, resulting in *NRXN1* methylation levels significantly exceeding those observed in control subjects (Jahn et al., 2021). Moreover, MIR-137 is a brain-enriched microRNA potentially implicated in the pathogenesis of schizophrenia (Mahmoudi and Cairns, 2017). Genome-wide DNA methylation profiling in schizophrenia revealed several differentially methylated genes (including *NRXN1*) are predicted targets of MIR-137 (Forero and González-Giraldo, 2020). Additionally, *NRXN1* also utilizes sex-specific methylation modifications and associated regulatory networks to modulate the sexually dimorphic susceptibility of neuropsychiatric disorders (Xia et al., 2021).

Abnormalities in alternative splicing also may contribute to schizophrenia (Sebastian et al., 2023). Advances in long-read sequencing have uncovered numerous novel transcript isoforms of schizophrenia-associated genes (Zhang et al., 2022). This underscores the potential role of alternative splicing in mediating genetic susceptibility, while also revealing the urgent need for expanded research efforts to systematically characterize these complex transcriptional profiles and their functional implications (Zhang et al., 2022).

The LNS4 domain of the NRXN1α isoform has been identified as a key region associated with the heritability of ASD and schizophrenia, in which three ultra-rare missense variants (T737M, D772G, and R856W) were found to be potentially pathogenic in these two disorders (Ishizuka et al., 2020). Functional characterization revealed that the D772G and R856W mutations significantly impaired the cell surface localization of NRXN1α, while both T737M and D772G variants exhibited diminished binding affinity to NLGN1 (Ishizuka et al., 2020).

Moreover, emerging evidence suggests NRXN1α may be involved in the association between schizophrenia and autoimmune dysregulation. Anti-NRXN1α autoantibodies are specifically identified in schizophrenia patients but absent in healthy controls (Shiwaku et al., 2023). At the molecular level, these autoantibodies disrupt the binding interactions between NRXN1α and both NLGN1 and NLGN2 (Shiwaku et al., 2023). At the level of neurons, these autoantibodies decreased mEPSC frequency and dendritic spine/synapse density in the murine frontal cortex (Shiwaku et al., 2023). In terms of behavior, behavioral phenotypes associated with schizophrenia were induced, such as decreased cognition, decreased social novelty preference and damaged pre-pulse inhibition (Shiwaku et al., 2023).

#### 5.1.2 Autism spectrum disorder

The ASD is a complex neurodevelopmental condition characterized primarily by impaired social interaction and stereotyped, repetitive behaviors or circumscribed interests (Hirota and King, 2023). A prevailing hypothesis regarding the etiology of ASD is the E-I imbalance in neural circuits of the brain, primarily involving excitatory glutamatergic neurons and inhibitory GABAergic neurons (Uzunova et al., 2016). Most E-I imbalance demonstrates the elevated excitation-inhibition ratio (E-I ratio) in the prefrontal cortex produces behavioral and social deficits characteristic of ASD, while a small number of ASD patients present reduced E-I ratio (Uzunova et al., 2016). Another hallmark of neural circuit organization in ASD is widespread long-range hypoconnectivity and disconnection, alongside localized hyperconnectivity (Uzunova et al., 2016).

Genome-wide screening for structural genomic variation in 427 unrelated ASD cases identified *NRXN1* as a factor contributing to ASD susceptibility, especially in terms of familial inheritance (Marshall et al., 2008). Whole-genome CNV analysis in the Autism Case–Control cohort (859 ASD cases and 1,409 healthy controls) and the Autism Genetic Resource Exchange cohort (1,336 ASD cases and 1,110 control subjects) both validated *NRXN1* as an ASD candidate gene (Glessner et al., 2009). Loss-of-function mutations in NRXN1 are among the main hereditary characteristics of ASD, associated with abnormal synaptic regulation and neural circuit development (Cooper et al., 2024). A recent study using the iPSYCH2015 case-cohort dataset found that individuals carrying exon-disrupting deletions in the NRXN1 gene had a 3-fold higher likelihood of ASD and a 2-fold higher risk of attention-deficit/hyperactivity disorder (ADHD) compared with non-carriers (Montalbano et al., 2024). However, most current genomic studies only focus on validating the role of *NRXN1* in ASD. Given that isoforms of NRXN1α (Yan et al., 2008; Williams et al., 2019) and NRXN1β (Feng et al., 2006) have been reported in individuals with ASD, future investigations should analyze the contribution ratio of NRXN1α and NRXN1β isoforms in this disorder.

The investigations related to the role of neurexin 1 in ASD are listed in Table 2. Functional characterization of three ASD-associated *NRXN1* missense mutations (Y282H, L893V, and I1135V) through *in vitro* and *Drosophila* models revealed impaired protein degradation kinetics relative to wild-type neurexin 1, resulting in slight functional perturbation of neurexin 1 activity (Liu et al., 2021). Additionally, *Adnp*, a high-risk ASD-associated gene, has recently been identified as an upstream regulator of *Nrxn1* (Wan et al., 2024). Following the knockdown of *Adnp*, the expression levels of *Nrxn1, Nlgn1, and Nlgn2* were markedly diminished and synaptic structural and functional deficits were observed (Grigg et al., 2020; Wan et al., 2024). The downregulation of NRXN1 disrupts the maturation of postsynaptic density (PSD) in dorsolateral prefrontal cortical circuits, which may be a key mechanism underlying synaptic abnormalities in ASD (Fatemi et al., 2024). Quantitative proteomic analysis of the dorsolateral prefrontal cortex identified impaired synaptic maturation patterns in patients with idiopathic autism, characterized by significant downregulation of ASD-associated risk genes involved in PSD development, including *NRXN1* (Fatemi et al., 2024). These molecular alterations were most pronounced during early neurodevelopment, while adult ASD cases exhibited less attenuated or unchanged PSD-related modifications, which corresponds to the identity of ASD as a neurodevelopmental disorder (Fatemi et al., 2024).

**Table 2:** Summary of functional studies of *NRXN1* in ASD.

Targeted mutation	Case sample	Control sample	Model/method	Effects/alterations	References
Missense mutation	16	16	*Drosophila* models with ASD-associated *Nrx1* missense mutations (Y282H, L893V, I1135V)	Synaptic activity:↓neurexin 1 degradation	Liu et al. (2021)
Deletion	3	9	*Nrxn1^+/−^* mice model	Synaptic activity:↓ADBE capability Behavior:↑ASD-related behaviors	Bonnycastle et al. (2025)
Alternative splicing	8–17	8–17	Mice model with insertion or knockout of *Nrxn1* SS5	Synaptic activity: in *Nrxn1* SS5 insertion, ↓neurexin 1 protein expression and glutamatergic transmission; in *Nrxn1* S5 exclusion mice, ↑neurotransmission Behavior: ↓ASD-related behaviors	Lu et al. (2023)
Isoforms	9–19	9–19	Mice model with mutant *Nrxn1β*	Synaptic activity:↓combination between beta-Nrxn1 and glutamatergic Nlgn1	Arias-Aragón et al. (2025)
Isoforms	83	42	Mice model with heterozygous loss of *Nrxn1α*	Behavior:↑ASD-related behaviors	Xu et al. (2023)
Isoforms	3	5	iPSCs from ASD patients carrying *NRXN1α*	Synaptic activity: ↑sodium currents and action potential amplitude, glutamatergic synaptic transmission and the activity of ion channels and transporters, particularly voltage-gated potassium channels	Avazzadeh et al. (2021)
Isoforms	20	19	Mice model with *Nrxn1* heterozygous deletion	Behavior: ↑learning of novel discrimination and latency to make correct choices	Hughes et al. (2022)
Isoforms	3	3	Bi-allelic *NRXN1α* deletion in microglia from iPSCs of ASD	Synaptic activity: ↑interleukin-6, a pro-inflammatory factor	Bose et al. (2025)

↑: increase; ↓: decrease.

**Figure 4:**
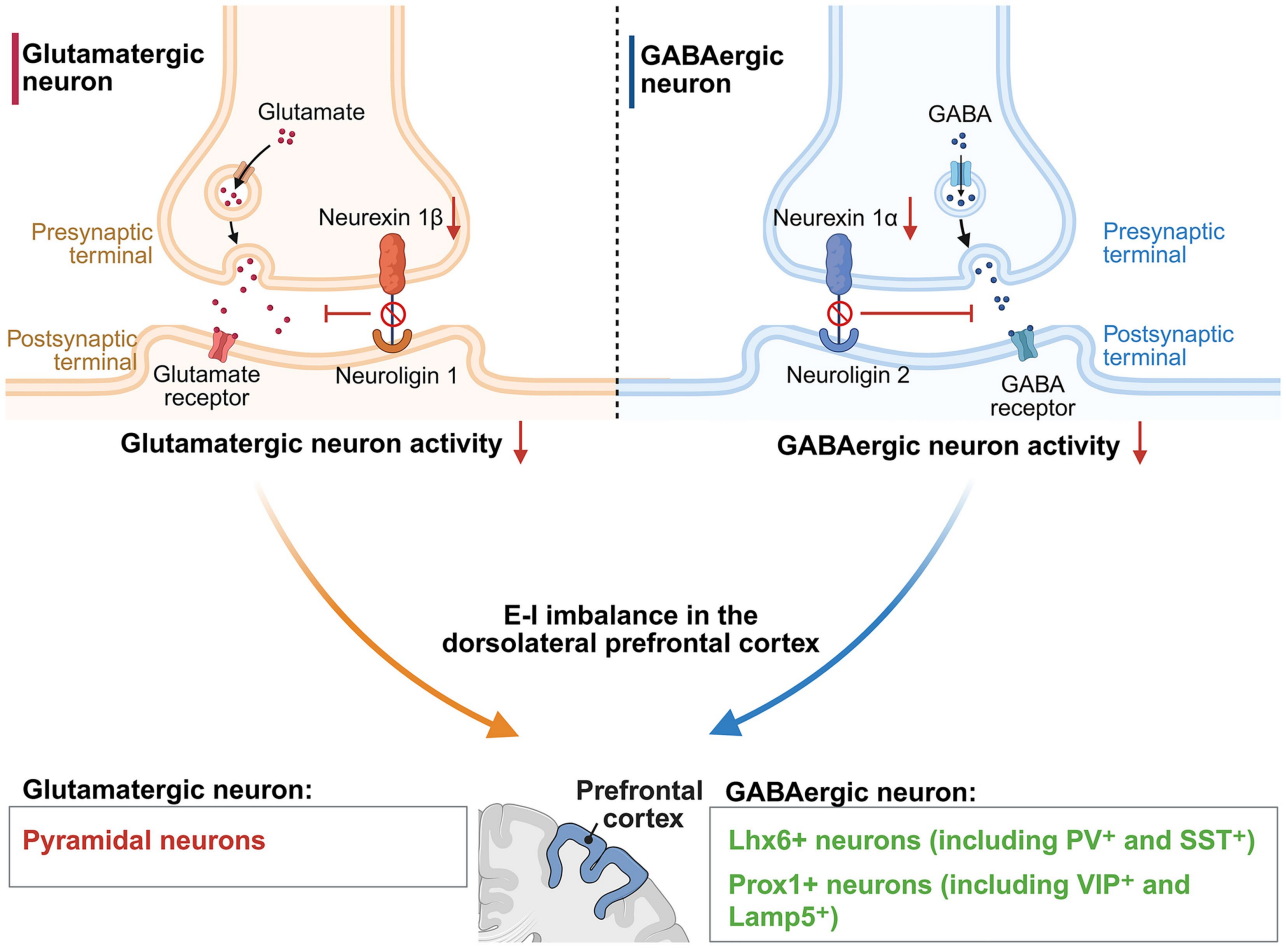
Isoform-dependent E-I imbalance hypothesis of *NRXN1* in autism spectrum disorder. In glutamatergic neurons, deficiency of neurexin 1β disrupts its interaction with neuroligin 1. This impairment consequently inhibits glutamate-mediated signal transduction, thereby reducing the activity of these excitatory neurons. In GABAergic neurons, deficiency of neurexin 1α disrupts its connection with neuroligin 2, resulting in reduced GABAergic signaling and activity of these inhibitory neurons. Diminished excitatory glutamatergic and inhibitory GABAergic activity both contribute to the E-I imbalance in the dorsolateral prefrontal cortex. This region contains excitatory glutamatergic neurons (including pyramidal neurons in layers 2–5) as well as diverse inhibitory GABAergic neurons: Lhx6 + neurons (PV^+^ and SST^+^ subclasses) and Prox1 + neurons (including VIP^+^ and Lamp5^+^ subclasses).

We herein postulate an isoform-dependent E-I imbalance hypothesis for the role of NRXN1 in ASD pathogenesis, depicted in Figure 4. Specifically, downregulation of NRXN1α impairs its interaction with GABAergic neuroligin 2, thereby reducing its ability to exert inhibitory control and elevating the E-I ratio. In contrast, downregulation of NRXN1β lowers the E-I ratio through reducing binding to glutamatergic neuroligin 1, thereby compromising glutamatergic neurotransmission in dorsolateral prefrontal cortex. Notably, most E-I imbalances in ASD are characterized by an elevated E-I ratio in the prefrontal cortex, while a small subset of ASD patients exhibits a reduced E-I ratio (Uzunova et al., 2016). This hypothesis may account for the divergent E-I imbalances observed in ASD patients. Therefore, we speculate that NRXN1α are more prevalent than NRXN1β in this disorder.

In an ASD mouse model, NRXN1α primarily functions as a presynaptic binding partner for GABAergic neuroligin 2, thereby mediating the activation of inhibitory GABAergic neurons (Arias-Aragón et al., 2025). Electrophysiological analyses of ASD pluripotent stem cell-derived *NRXN1α^+/−^* cortical neurons revealed significantly elevated neuronal excitability, characterized by amplified sodium currents, elevated action potential amplitude, and accelerated depolarization kinetics (Avazzadeh et al., 2021). These alterations were correlated with a marked upregulation of glutamatergic synaptic activity and ion channel activity, particularly voltage-gated potassium channels (Avazzadeh et al., 2021). At the organ level, attenuated *Nrxn1α* expression in mice significantly impairs prefrontal cortical function, leading to impaired cognitive flexibility (Hughes et al., 2022). At the behavior level, Xu et al. observed that mice with heterozygous deletion of *Nrxn1α* exhibited altered social novelty preference and repetitive motor behaviors, which are similar to the two characteristics of autism: deficits in social interaction and restricted, repetitive behavioral patterns (Xu et al., 2023). In an ASD patient-derived iPSC *in vitro* model, bi-allelic deletion of *NRXN1α* damages both neuronal and microglial function (Bose et al., 2025). Microglia release interleukin-6 (IL-6) and are impaired in sustaining the formation of functional neuronal circuitry (Bose et al., 2025). Notably, IL-6 level is significantly enhanced in the cerebellum of ASD patients (Wei et al., 2011). Increased IL-6 may disrupt neural cell adhesion and migration, and drive the E-I imbalance (Wei et al., 2011). Thus, in addition to neurons, microglia are also adversely affected by bi-allelic deletion of *NRXN1α* and may contribute to the pathogenesis of ASD.

Relative to NRXN1α, NRXN1β has been involved in the pathogenesis of ASD through distinct molecular mechanisms, primarily involving disrupted connectivity with neuroligin 1. The beta-Nrxn1ΔC mouse model recapitulates partial characteristics of ASD, in which the expression of a C-terminally truncated NRXN1β mutant (lacking SS4) in adult forebrain neurons results in a selective impairment of the NRXN1β/neuroligin 1 signaling pathway (Arias-Aragón et al., 2025). Although NRXN1β can interact with neuroligin 2 (Ullrich et al., 1995), these findings indicate that mutated NRXN1β selectively binds neuroligin 1 in ASD, underscoring that NRXN1β predominantly mediates excitatory signaling. And these results are consistent with the previous experiment: following NRXNβ knockout, excitatory synapses in cultured cortical neurons exhibited severe impairment of neurotransmitter release (Anderson et al., 2015). Therefore, in contrast to NRXN1α, disease-associated mutation in NRXN1β isoforms disrupts the E-I balance by diminishing synaptic excitation at glutamatergic synapses (Arias-Aragón et al., 2025).

In addition to SS4, SS5 has also been implicated in the pathogenesis of ASD (Lu et al., 2023). The insertion of SS5 enhanced heparan sulfate chain formation, a post-translational modification, followed by diminished NRXN1 protein expression and glutamatergic neurotransmitter secretion (Lu et al., 2023). In turn, genetic exclusion of SS5 in murine models enhances spontaneous and evoked transmission, hippocampal excitatory synapse density, presynaptic release frequency, while displaying no alterations at inhibitory synapses (Lu et al., 2023). Nevertheless, NRXN1α and NRXN1β isoforms were not separately discussed in this investigation.

Emerging findings have uncovered novel insights into the ASD’s pathogenic role of *Nrxn1* in mediating the impairment of synaptic vesicle endocytosis and complex interaction with other nuclear and mitochondrial genetic variants. Activity-dependent bulk endocytosis (ADBE) serves as the predominant synaptic vesicle retrieval pathway under conditions of high neuronal activity (Kim et al., 2024). In *Nrxn1+/−* rats, ADBE capacity is significantly impaired, indicating the low neuronal activity (Bonnycastle et al., 2025). Nevertheless, this investigation was restricted to primary hippocampal neuron cultures, leaving the potential effects on other neuronal populations and modified neural networks unexplored (Bonnycastle et al., 2025). In addition to the dysfunction of synaptic vesicle endocytosis, the interplay of rare genomic variants across nuclear and mitochondrial genomes collectively influences disease pathogenesis. In an ASD pedigree with inherited *NRXN1* deletion, the affected child exhibited a higher burden of exonic rare variants compared to the unaffected mother carrying the same deletion (Cameli et al., 2021). Protein–protein interaction analysis revealed significant enrichment among genes carrying deleterious variants in the proband, implying that co-occurring rare variants in functionally related genes may synergistically exceed the pathogenicity threshold for ASD development (Cameli et al., 2021). Moreover, mitochondrial DNA analysis identified five novel low-level heteroplasmic variants present exclusively in the proband, along with two maternally inherited variants with elevated heteroplasmic loads, indicating synergies between maternally derived mitochondrial genes and rare nuclear genes, such as *NRXN1* (Cameli et al., 2021).

Notably, not all categories of ASD mouse models demonstrate the downregulation of *Nrxn1*. Simmons et al. reported the upregulated expression of *Nrxn1* in the *patDp/+* mouse model, which mimicked the human 15q11-13 duplication, one of the ASD etiologies, exhibiting pathological amplification of climbing fiber signaling (Simmons et al., 2022). This neural dysregulation likely stems from *Nrxn1* overexpression, ultimately disrupting sensory encoding by climbing fiber inputs and impairing parallel fiber synaptic architecture and plasticity (Simmons et al., 2022). Such mechanistic alterations may underlie the characteristic sensory processing abnormalities and motor coordination deficits observed in ASD (Simmons et al., 2022). A significant constraint of the current investigation is the limited sample size (*n* = 3 per group) in both the experimental and control mouse cohorts (Simmons et al., 2022).

Recently, a novel analytical framework has been developed to potentiate diagnostic precision for neuroimaging of *NRXN1* mutation-related ASD. Singh et al. developed an integrative analytical framework that combines neurite orientation dispersion and density index diffusion magnetic resonance imaging with radiomics feature extraction (Singh et al., 2023). This novel approach enables precise differentiation among four genetically defined ASD rat models (*Nrxn1*, *Fmr1*, *Pten*, and *Disc1*), potentially reducing the neurobiological heterogeneity challenges inherent in ASD research (Singh et al., 2023).

#### 5.1.3 Attention-deficit hyperactivity disorder

The ADHD is a common neurodevelopmental disorder, characterized by inattention, hyperactivity, and impulsiveness that are inconsistent with developmental level (Koirala et al., 2024). This disorder represents the most prevalent co-occurring condition in children diagnosed with ASD, with estimated comorbidity rates ranging from 40 to 70% (Antshel and Russo, 2019). Additionally, ADHD shares multiple overlapping CNVs with both schizophrenia and ASD, including deletions at the 2p16.3 locus (*NRXN1*) (Gudmundsson et al., 2019).

The impacts of neurexin 1 in ADHD are depicted in Figure 3B. Zhang et al. (2021) utilizing the ADHD rat model demonstrated deficiency of *Nrxn1* contributes to ADHD pathogenesis, manifesting as impaired learning and memory ability. Their study further revealed that reduced *Nrxn1* expression dysregulates multiple synapse-related proteins, including PSD95, SYN1, GAP43, and NLGN1, thereby disrupting learning- and memory-associated signaling pathways in ADHD (Zhang et al., 2021). In turn, overexpression of *Nrxn1* led to elevated expression of synapse-related genes at both the mRNA and protein levels, corresponding with improved learning and memory in ADHD rat model (Zhang et al., 2021). Another ADHD rat model experiment revealed downregulation of *Nrxn1* is involved in MALAT1-miR-141–3p/200a-3p-Nrxn1 axis to decrease synapse-related proteins SYN1, PSD95, and GAP43, eventually impairing the memory and learning capacity in ADHD rats (Mu et al., 2023). These studies suggest the possible neuroprotective role of neurexin 1 in ADHD-related cognitive processes.

#### 5.1.4 Insomnia

Insomnia represents a widespread sleep disorder manifesting as impaired sleep initiation or continuity, which contributes to significant health deterioration, elevated mortality risk, and considerable socioeconomic impacts (Sutton, 2021). Bioinformatics-based gene network analysis identified *NRXN1* as a particularly promising therapeutic target for insomnia, supported by its robust functional annotation score and documented pharmacological interactions with both duloxetine hydrochloride and nicotine polacrilex (Satria et al., 2025).

The impacts of *NRXN1* in insomnia are indicated in Figure 3C. Heterologous expression of human *NRXN1* in *Drosophila melanogaster* may disrupt circadian regulation and induce sleep pattern abnormalities (Liu et al., 2021). Moreover, a novel semi-automated scoring system employed frequency-specific profiles to classify vigilance states across sleep–wake cycles, establishing an optimized electroencephalographic analytical framework for detecting translational biomarkers associated with sleep architecture and excitation-inhibition balance in neuropsychiatric disorders (Østergaard and Kas, 2025). Application of this methodology in *Nrxn1α*-deficient mice revealed a significant attenuation in slow-wave sleep duration relative to wild-type controls (Østergaard and Kas, 2025), providing functional evidence that *Nrxn1α* mutations contribute to the pathophysiology of insomnia. However, comprehensive experimental studies remain imperative to fully characterize the mechanistic role of *NRXN1* in insomnia pathophysiology and treatment.

#### 5.1.5 Epilepsy

Epilepsy patients harboring *NRXN1* microdeletions frequently exhibit generalized seizure phenotypes and psychiatric comorbidities, with exon 1–6 deletions representing a predominant genetic lesion particularly prevalent in genetic generalized epilepsy cohorts (Guo et al., 2025). The brains of epilepsy patients prevalently exhibit exceptionally high metabolic demands, necessitating dynamic bioenergetic regulation to maintain physiological function while compensating for pathological energy requirements during spontaneous recurrent seizures (Rho and Boison, 2022). Disruptions in cellular or mitochondrial metabolic homeostasis may serve as primary triggers for epileptogenesis, ultimately contributing to the development of spontaneous recurrent seizure activity (Rho and Boison, 2022).

The impacts of *NRXN1* in epilepsy are indicated in Figure 3D. In a *Drosophila melanogaster* model, genetic deletion of *Nrx1* induces metabolic dysregulation and enhanced seizure susceptibility, corresponding with mitochondrial dysfunction (Levy et al., 2022). Additionally, the destabilization of synaptic integrity compromises electrophysiological neurotransmission, constituting the fundamental electrophysiological mechanism in epilepsy. For instance, perturbations in nanoscale spatial coordination between NRXN1 and the postsynaptic scaffold protein PSD-95 induce maldistribution of synaptic components, reflecting compromised synaptic integrity and contributing to neuronal hyperexcitability with epilepsy characteristics. These mechanistic insights necessitate further validation through clinical investigations involving epilepsy patient cohorts (Fukata et al., 2021).

#### 5.1.6 Suicide

Suicide encompasses two distinct yet interrelated components: suicidal ideation and suicidal behavior (Gonda et al., 2023). Globally, this devastating phenomenon claims nearly 700,000 lives annually, representing a significant public health crisis (Gonda et al., 2023). Emerging genome-wide evidence indicates that *NRXN1*, particularly two non-synonymous variants (rs78540316; P469S and rs199784139; H885Y), contributes to familial susceptibility to suicide (William et al., 2021).

The impacts of *NRXN1* in suicide are indicated in Figure 3E. Mechanistic investigations further demonstrate that while the P469S variant of *NRXN1* does not affect hemi-synapse formation compared to the wild-type, both variants exhibit enhanced *in vitro* binding affinity for the postsynaptic interactor leucine-rich repeat transmembrane neuronal 2 (LRRTM2) (William et al., 2021). Moreover, a recent GWAS highlighted the NLGN1 locus surpasses genome-wide significance thresholds for suicide mortality (Li et al., 2023). Therefore, the NRXN1-NLGN1 signaling pathway emerges as a potential therapeutic target for suicide prevention.

#### 5.1.7 Depression

Depression represents the predominant psychiatric manifestation encountered in primary healthcare practice (McCarron et al., 2021). Synaptic plasticity alterations represent fundamental downstream mechanisms mediating persistent antidepressant responses, highlighting the possible role of SAMs in depression (Ruan et al., 2024). Currently, research exploring the association between depression and NRXN1 remains at a preliminary stage. A pilot study demonstrated that both *NRXN1* mRNA expression and NRXN1 protein levels were significantly lower in patients with depression than in healthy controls (Skiba et al., 2021). And most investigations focus on NRXN2 and NRXN3. A proteomic analysis revealed a significantly decreased NRXN3 protein expression level in the cerebrospinal fluid of 40 patients with major depressive disorder (Al Shweiki et al., 2020). Early life stress elevates the risk of depression in children and adolescents (LeMoult et al., 2020).

In a rat stress model, postnatal maternal separation stress increases susceptibility to restraint stress in adulthood (Rong et al., 2025). The NRXN3-NLGN1 complex has been shown to be involved in this stress-induced susceptibility process (Rong et al., 2025). Moreover, knockdown of *St3gal1*—a sialyltransferase involved in O-glycosylation—induced depressive-like symptoms in non-stressed mice, including reduced motivation to feed and weight loss, whereas its overexpression in stressed mice attenuated these symptoms (Seo et al., 2025). Additionally, researchers identified NRXN2 as a potential target and downstream regulator of St3gal1 (Seo et al., 2025).

#### 5.1.8 Tourette syndrome, anorexia nervosa, trichotillomania, and intellectual disability

Pathogenic variants in *NRXN1* have been further implicated in diverse neuropsychiatric disorders, including Tourette syndrome, anorexia nervosa, trichotillomania, and intellectual disability, though these relationships require further confirmation. Genome-wide interrogation of low-frequency CNVs (population prevalence <1%) through Single Nucleotide Polymorphisms (SNPs) microarray profiling identified statistically significant associations of both *NRXN1* deletions and *CNTN6* duplications with the predisposition of Tourette syndrome, a neurodevelopmental condition demonstrating strong heritability (Huang et al., 2017). Moreover, the first large-scale GWAS of rare CNV analysis in anorexia nervosa, a severe psychiatric condition marked by starvation and nutritional deficiency (Thavaraputta et al., 2023), revealed the nominal association between *NRXN1* intron 5 (2p16.3) deletion and disease susceptibility (Walker et al., 2025). Furthermore, trichotillomania, characterized by compulsive hair-pulling behavior, leads to clinically significant alopecia and psychological impairment (Grant et al., 2023). Trichotillomania has been associated with genomic deletions in *NRXN1* through molecular genetic analysis (Halvorsen et al., 2025). Additionally, whole-exome sequencing revealed *NRXN1* deletions as a genetic contributor to non-syndromic intellectual disability (Taşkıran et al., 2021). Thus, further investigations are warranted to clarify the precise mechanistic contributions of *NRXN1* in these neuropsychiatric disorders.

### 5.2 Neurodegenerative disorders

Emerging evidence implicates synaptic dysfunction as a critical pathological mechanism underlying neurodegenerative disorders, including Huntington’s disease (HD) and Parkinson’s disease (PD), which profoundly compromise patients’ quality of life. Notably, altered NRXN1 expression level has been identified as a key contributor to synaptic impairment across multiple neurodegenerative conditions (Cho and Kim, 2025). Moreover, recent studies indicate that both neurosteroids and growth factors may exert neuroprotective effects by ameliorating NRXN1-mediated synaptic regulation (Kraus et al., 2013; Sheibani et al., 2022).

Huntington’s disease, a neurodegenerative condition mediated by mutant huntingtin (HTT) protein, is characterized by progressive synaptic impairment and neuronal loss. Notably, both HD mouse models at symptomatic stages and polyQ-expanded neuronal cultures exhibit marked downregulation of *Nrxn1* expression (Cho and Kim, 2025). Cho and Kim (2025) revealed that mutant HTT enhanced the expression of lysosome-associated membrane protein 2A (LAMP2A), activating the lysosomal pathway which degraded NRXN1. These results underscore the pivotal role of NRXN1 in Huntington’s disease-associated synaptic impairment and suggest that modulating LAMP2A may be an approach to mitigate disease pathogenesis.

Similarly, in PD, dopaminergic neuron degeneration similarly leads to disrupted synaptic plasticity. 6-hydroxydopamine (6-OHDA)-induced PD rat models demonstrated concomitant NRXN1 downregulation and disruption of long-term potentiation (LTP) in the hippocampus (Sheibani et al., 2022). Notably, the neurosteroid allopregnanolone (Allo) ameliorated these pathological alterations, normalized NRXN1 expression, and enhanced synaptic transmission (Sheibani et al., 2022). These results demonstrate NRXN1’s pivotal involvement in PD development while revealing Allo’s potential neuroprotective effects mediated through NRXN1 expression regulation.

### 5.3 Neurological tumors

Glioblastoma multiforme (GBM) is recognized as the most prevalent primary brain tumor in adults (Fabian et al., 2019). This malignancy is highly aggressive and infiltrative, leading to an extremely poor prognosis with almost universal recurrence (Uthamacumaran et al., 2025). Even with aggressive treatment, the median overall survival is approximately 14.6 months (Feng et al., 2022; Uthamacumaran et al., 2025). Two novel NRXN1-targeting therapies for GBM have been developed, specifically immunotherapy and exogenous NRXN1 therapy. Recent research indicated that exogenous NRXN1 influences tumor cell fate, suppresses malignant phenotypes, and is associated with improved prognosis in GBM (Feng et al., 2022; Liao et al., 2024). In Liao’s study, researchers developed multiple *in vivo* models, such as orthotopic xenograft glioma and subcutaneous xenograft tumor models (Liao et al., 2024). Their findings demonstrated that exogenous NRXN1 protein supplementation significantly improved glioblastoma prognosis, an effect attributed to promoting tumor cell differentiation toward neuronal lineages (Liao et al., 2024). This process was further associated with NRXN1-mediated downregulation of genes within the AP1 complex (Liao et al., 2024).

Immunotherapy investigations have demonstrated that gene clusters containing *NRXN1*, along with genes such as *KCNIP2*, *SCRT1*, and *MAP2*, serve as a characteristic gene expression signature for the immunologically cold phenotype in glioblastoma (Feng et al., 2022). Therefore, high expression of NRXN1 may act as a molecular biomarker indicating that glioblastoma exhibits an immunologically suppressed “cold” phenotype. Consequently, *NRXN1* gene may be associated with an immunosuppressed tumor microenvironment and poor clinical prognosis (Feng et al., 2022). Whether the *NRXN1* gene serves as the principal regulator within this gene cluster, and why exogenous NRXN1 protein overexpression correlates with improved prognosis, whereas high endogenous *NRXN1* gene expression predicts poor outcomes, necessitates further investigation.

Neuroblastoma (NB), a pediatric malignancy, originates from neural crest cells (Qiu and Matthay, 2022). This tumor can arise in any site containing sympathetic ganglia or the adrenal medulla (Qiu and Matthay, 2022). NB cell clones with stable shRNA-mediated knockdown of human NRXN1α exhibit enhanced tumor growth (Fanlo et al., 2023). Downregulation of NXPH1 expressions induced the shift from an adrenergic to a mesenchymal state in NB cells (Fanlo et al., 2023). Furthermore, NRXN1α also suppressed organ-specific colonization and metastasis, establishing the NRXN1α as a potential therapeutic target for high-risk neuroblastomas (Fanlo et al., 2023). Consequently, NRXN1 modulates neuroblastoma cell states and drives neuroblastoma progression. Nevertheless, the study only highlights the significant role of NRXN1α in this network.

## 6 *NRXN1*-targeted treatment for neuropsychiatric disorders

The exclusion of SS5 represents a promising therapeutic strategy for neuropsychiatric disorders associated with NRXN1 deficiencies. Recently, Lu et al. (2025) revealed the therapeutic potential of SS5 exclusion in *Nrxn1+/−* mice. Excising the SS5 segment from the remaining *Nrxn1* allele successfully mitigated synaptic and behavioral deficits. Specifically, this intervention restored miniature excitatory postsynaptic current frequency, paired-pulse ratio, AMPA/NMDA ratio, and repetitive behaviors to wild-type levels, while partially rescuing NRXN1 protein expression in *Nrxn1*ΔSS5/− mice compared to *Nrxn1+/−* controls (Lu et al., 2025).

Furthermore, SNPs in *NRXN1* have been associated with treatment outcomes of antipsychotic agents. In a double-blind, placebo-controlled crossover trial involving 54 schizophrenia inpatients, Jenkins et al. investigated the association of two *NRXN1* SNPs (rs12467557 and rs10490162) with antipsychotic treatment response (Jenkins et al., 2014). Their pharmacogenetic analysis revealed that patients homozygous for the A allele at either SNP exhibited significant improvement across multiple symptom domains, including positive, negative, and general psychopathology, particularly in thought disturbance. In contrast, carriers of the G allele showed no significant response (Jenkins et al., 2014). Although subsequent analysis in the larger CATIE trial cohort did not replicate these specific SNP associations, it identified weakly positive signals at other *NRXN1* loci (Jenkins et al., 2014). Collectively, these results support the role of *NRXN1* genetic variation in mediating differential treatment outcomes to antipsychotic drugs among patients with schizophrenia, thereby implicating this gene in the therapeutic mechanism of antipsychotic agents.

Recently, Duloxetine Hydrochloride and Nicotine Polacrilex have been identified as promising *NRXN1*-trageting antipsychotic agents. An investigation of a drug-gene interaction database for compounds targeting *NRXN1* identified Duloxetine Hydrochloride and Nicotine Polacrilex (Satria et al., 2025). Given their specific interactions with *NRXN1* and functional annotation score of *NRXN1* for insomnia, these drugs are proposed as possible candidates for the treatment of *NRXN1*-associated insomnia (Satria et al., 2025).

## 7 Discussion

In this review, we provide an overview of the association between neurexin 1 and neuropsychiatric disorders, especially schizophrenia and ASD, suggesting *NRXN1* as a potential pharmacological target for neuropsychiatric disorders. In schizophrenia, multiple genetic and epigenetic alterations of *NRXN1*—including CNVs, methylation status, and alternative splicing patterns— may contribute to disease pathogenesis. In ASD, downregulation of NRXN1, arising from genetic deletions and aberrant alternative splicing, may compromise key synaptic processes, including synaptogenesis and activity-dependent bulk endocytosis. Additionally, *NRXN1* has also been associated with some other neuropsychiatric conditions, while the precise involvement of *NRXN1* in these disorders warrants further validation. Beyond its well-established role in psychiatric disorders, *NRXN1* attracts the interest of researchers in other fields, including neurodegenerative diseases and neurological oncology.

We proposed the isoform-dependent E-I imbalance hypothesis of NRXN1 in ASD. Specifically, downregulation of NRXN1α elevates the E-I ratio by impairing its interaction with GABAergic neuroligin 2, while NRXN1β downregulation reduces the E-I ratio through decreasing binding to glutamatergic neuroligin 1. Our hypothesis bridges the specific ligand interactions of NRXN1α and NRXN1β with ASD pathogenesis. This hypothesis provides a feasible explanation for the diverse E-I imbalance in patients with ASD. Nevertheless, we concede that our hypothesis has some limitations. The downregulation models of NRXN1α and NRXN1β were not the same: NRXN1α downregulation utilized an ASD pluripotent stem cell-derived *NRXN1*α^+/−^ cortical neurons model, while NRXN1β downregulation utilized a beta-Nrxn1ΔC mouse model. Therefore, subsequent investigations should employ the same experimental model to maintain consistency and control for variables.

Nevertheless, several challenges constrain the investigation of both isoforms within one single model system. The two isoforms exhibit distinct expression patterns across different cell populations: NRXN1α is enriched in cortical glutamatergic pyramidal neurons, while NRXN1β is preferentially expressed in GABAergic PV^+^ interneurons (Nguyen et al., 2016). This brings a challenge for simultaneously mimicking the physiological conditions of both isoforms. Notably, human brain organoids derived from pluripotent stem cells offer a promising alternative by recapitulating the three-dimensional architecture of the human brain from neuropsychiatric disorders individuals. A critical technical milestone in this field will be the development of a single model system (e.g., human brain organoids) that allows for the simultaneous manipulation of all isoforms of one molecule. For instance, utilizing this model system, researchers can apply gene-editing approaches to investigate how neurexin 1 isoforms collectively modulate neuronal firing rates in glutamatergic and GABAergic neurons of ASD patient-derived organoids.

Additionally, when validating the hypothesis, the potential influence of alternative splicing and overlapping gene architecture of *NRXN1α* and *NRXN1β* (such as SS4 and SS5) should be considered, as these factors may lead to off-target effects and interfere with isoform-specific manipulation. In the investigation of *Nrxn1β* function in ASD, the beta-Nrxn1ΔC mouse model harbors a deletion of the SS4 splicing cassette (Arias-Aragón et al., 2025). As this cassette is conserved in *Nrxn1α*, its deletion may also disrupt the transcription of *Nrxn1α*, confounding result interpretation. Thus, future work requires the development of alternative approaches for isoform-specific targeting of neurexin 1β, with no off-target effects on the expression or function of neurexin 1α. In addition to SS4, the role of the SS5 splicing cassette also merits further attention: prior work in murine models has shown that SS5 exclusion increases excitatory synapse density and neuronal firing, with no effects on inhibitory synapse function (Lu et al., 2023). Accordingly, future studies should separately investigate the functional impact of SS5 splicing in the context of both isoforms.

Interestingly, both schizophrenia and ASDs involve deletions and alternative splicing events affecting *NRXN1*, offering molecular evidence for their comorbidity (the common feature among neuropsychiatric disorders). Although various alterations in the *NRXN1* gene contribute to the pathogenesis of neuropsychiatric disorders, most studies to date have primarily focused on establishing associations between *NRXN1* variants and disease susceptibility rather than elucidating the underlying molecular mechanisms. Therefore, future research should prioritize delineating the specific signaling pathways through which *NRXN1* dysfunction influences the pathophysiology of neuropsychiatric disorders. Moreover, human brain organoids derived from pluripotent stem cells offer a promising alternative by recapitulating the three-dimensional architecture of the human brain in individuals with neuropsychiatric disorders. Thus, future investigations should prioritize the use of human-based models—such as brain organoids or other human cellular systems—to further elucidate the role of *NRXN1* in neuropsychiatric disorders.

Cutting-edge technologies, including nanotechnology and deep learning, have largely propelled the development of novel approaches to explore NRXN1 biosynthesis, trafficking mechanisms, and structural characterization. The development of a nanobody-based imaging strategy has significantly advanced *in vivo* visualization of NRXN1 dynamics. Vicidomini et al. (2024) employed an ALFA tag (AT)/nanobody system to reveal that the PSD zone-binding motif critically regulates the synaptic targeting and functional integrity of Nrx1 in *Drosophila*. Furthermore, their methodology enabled spatially resolved detection of endogenous Nrx1, real-time monitoring of its axonal transport in motor neurons, and demonstration of its co-trafficking with Rab2-associated vesicular compartments (Vicidomini et al., 2024). Moreover, Rhoades et al. (2022) utilized computational saturation mutagenesis analysis combined with AlphaFold, a deep learning-based protein structure prediction tool revealed that *NRXN1* mutations related to schizophrenia and ASD likely disrupt protein stability, thereby demonstrating a direct structure–function correlation in this synaptic protein.

Advances in statistical methodologies for GWAS have enhanced the diagnosis of *NRXN1* mutation-associated neuropsychiatric disorders and patient stratification based on *NRXN1* variants, accelerating the implementation of precision medicine guided by genetic profiling. KnockoffTrio—a novel statistical framework specifically designed for trio-based GWAS—demonstrates superior capability in identifying putative causal variants (Yang et al., 2022). When applied to ASD cohorts, this approach uncovers significant associations at the *NRXN1* locus that are commonly missed by conventional methods (Yang et al., 2022).

Advancing precision medicine approaches for *NRXN1*-related pathologies will necessitate patient stratification based on the mechanistic underpinnings of *NRXN1* mutations (loss-of-function and gain-of-function), enabling tailored interventions to either restore wild-type isoform expression or suppress pathogenic variants (Fernando et al., 2025).

In the future, elucidating the precise molecular mechanisms by which *NRXN1* contributes to neuropsychiatric disorders will necessitate the integration of advanced multidisciplinary approaches, including brain organoids, nanotechnology, deep learning, and sophisticated statistical modeling, thereby propelling the advancement of precision medicine approaches to *NRXN1*-related pathologies.
